# Supplementary material for: Job burnout among Israeli healthcare workers during the first months of COVID-19 pandemic: The role of emotion regulation strategies and psychological distress
Source: PLoS One. 2022 Mar 24;17(3):e0265659. doi: 10.1371/journal.pone.0265659 (PMC8947073; doi:10.1371/journal.pone.0265659)
Supplement: Attachment — Submitted filename: Response to Reviewers.docx [file pone.0265659.s002.docx]

February 26, 2022

Dear Prof. van Amelsvoort,
Academic Editor
PloS one

Thank you for offering us the opportunity to revise and resubmit our paper entitled “Job Burnout Among Israeli Healthcare Workers During First Months of COVID-19 Pandemic: The Role of Emotion Regulation Strategies and Psychological Distress” to PLOS ONE. We have added the details requested by Reviewer 1 and hope that the manuscript is now suitable for publication in PLOS ONE.

Sincerely,

Marlyn Khouri, Dana Lassri, and Noga Cohen.

**Comments of Reviewer #1**

I thank the authors for taking my comments into consideration and for revising the manuscript so thoroughly. I believe the current version is much improved. A few minor comments about the results section:

1. Please refer to R square as 0.02, and not 2%.

2. When reporting the steps of the hierarchical regression, please include the R square change value, which is crucial to get an idea about your effect size.

**Response: We thank the reviewer for the time and effort he invested in reviewing our manuscript. We changed the R^2^ value to 0.02 and also added details about R^2^ change.**

Page 13: “Burnout scores served as dependent variable. The first step, which included age and gender, did not account for significant variance in burnout, *R^2^* = 0.02, *F*(2, 95) = .72, *p* = .49. The second step, in which psychological distress and COVID-19 concerns were added, was significant, *F*(4, 93) = 15.58, *p* < .001, and accounted for 40% of the variance in burnout. This step added significantly to the model, accounting for an additional 39% of the variance in burnout score, *F*change (2, 93) = 30.00, *p* < .001. In this step, only psychological distress (total DASS score) significantly predicted burnout (β = .60, *t* = 6.21, *p* < .001). The third step, which included also emotion regulation strategies, was also significant, *F*(7, 90) = 10.97, *p* < .001, and accounted for 46% of the variance in burnout. This step added significantly to the model, accounting for an additional 6% of the variance in burnout score, *F*change (3, 90) = 3.30, p < .05. In this step, only worry (β = .24, *t* = 2.57, *p* < .05) and psychological distress (β = .49, *t* = 4.71, *p* < .001) significantly predicted burnout.”
